# NatalieQ: A web server for protein-protein interaction network querying

**DOI:** 10.1186/1752-0509-8-40

**Published:** 2014-04-01

**Authors:** Mohammed El-Kebir, Bernd W Brandt, Jaap Heringa, Gunnar W Klau

**Affiliations:** 1 Life Sciences, Centrum Wiskunde & Informatica, Science Park 123, 1098 XG Amsterdam, the Netherlands; 2 Centre for Integrative Bioinformatics VU (IBIVU), VU University Amsterdam, De Boelelaan 1081A, 1081 HV Amsterdam, the Netherlands; 3 Amsterdam Institute for Molecules, Medicines and Systems (AIMMS), Amsterdam, the Netherlands; 4 Department of Preventive Dentistry, Academic Centre for Dentistry Amsterdam (ACTA), University of Amsterdam and VU University Amsterdam, Amsterdam, the Netherlands; 5 Netherlands Bioinformatics Centre, Geert Grooteplein 28, 6525 GA Nijmegen, the Netherlands

**Keywords:** Network alignment, Protein-protein interaction, Sequence similarity, Topology, Wnt signaling pathway

## Abstract

**Background:**

Molecular interactions need to be taken into account to adequately model the complex behavior of biological systems. These interactions are captured by various types of biological networks, such as metabolic, gene-regulatory, signal transduction and protein-protein interaction networks. We recently developed Natalie, which computes high-quality network alignments via advanced methods from combinatorial optimization.

**Results:**

Here, we present NatalieQ, a web server for topology-based alignment of a specified query protein-protein interaction network to a selected target network using the Natalie algorithm. By incorporating similarity at both the sequence and the network level, we compute alignments that allow for the transfer of functional annotation as well as for the prediction of missing interactions. We illustrate the capabilities of NatalieQ with a biological case study involving the Wnt signaling pathway.

**Conclusions:**

We show that topology-based network alignment can produce results complementary to those obtained by using sequence similarity alone. We also demonstrate that NatalieQ is able to predict putative interactions. The server is available at: http://www.ibi.vu.nl/programs/natalieq/.

## Background

To adequately model complex behavior of biological systems one needs to take molecular interactions into account. These interactions are captured by various types of biological networks such as metabolic, gene-regulatory, signal transduction and protein-protein interaction (PPI) networks. Recent advances in technological developments and computational methods have resulted in large amounts of network data. For instance, STRING [1], a database of experimentally verified and computationally predicted protein interactions, grew from 261,033 proteins in 89 organisms in 2003 to 5,214,234 proteins in 1,133 organisms in January 2014. However, the development of solid methods for analyzing network data is lagging behind, particularly in the field of comparative network analysis. Here, one wants to detect commonalities between biological networks from different strains or species, or derived from different conditions. In contrast to traditional comparison at sequence level, topology-based comparison methods explicitly take interactions into account and are thus more suitable to compare networks. Subnetworks with shared interactions across species allow for improved transfer of functional annotations from one species to the other by using more information than sequence alone [2].

We have developed NATALIEQ, a web server for accurate topology-based protein-protein interaction network queries. It provides an interface to the general network alignment method NATALIE[3,4], which is fast and supports various scoring schemes taking both node-to-node correspondences and network topologies into account. Briefly, NATALIE views the network alignment problem as a generalization of the well-studied quadratic assignment problem and solves it using techniques from integer linear programming.

Currently, only few web servers for comparative network analysis exist. The PathBLAST web server [5] reports exact and approximate hits in a target PPI network for a user-defined simple query, expressed as a linear path of up to five proteins. The NetworkBLAST web server [6] finds locally-conserved protein complexes between species-specific PPI networks. NetAligner [7], a recent web server, allows the comparison of user-defined networks or whole interactomes within a set of fixed species using a heuristic network alignment with no guarantees on the optimality of the identified solutions.

Our contribution is twofold. First, NATALIEQ employs a new scoring function to produce high-quality pairwise alignments between a user-specified query network of arbitrary topology and interactomes of several model species and human. The score of an alignment is primarily based on the number of conserved interactions, while sequence similarity is used as a secondary, subordinate optimization goal. In addition, the alignments computed by the underlying NATALIE algorithm come with a quality guarantee that often proves their optimality. Second, through an interactive visualization of the alignment, the user can quickly get an overview of conserved and non-conserved interactions and can use the protein descriptions of the nodes to assess the alignment. We illustrate a usage scenario of the web server on the Wnt signaling pathway and demonstrate that NATALIEQ is able to predict putative interactions that are not detected by other methods.

## Implementation

### Network alignment algorithm

NATALIE, the alignment method of NATALIEQ, is applicable to any type of network and supports any additive score function taking both node-to-node correspondences and topology into account. Here, we take as input a pair of PPI networks whose nodes and edges correspond to proteins and their interactions. Let *G*_1_ = (*V*_1_,*E*_1_) and *G*_2_ = (*V*_2_,*E*_2_) be two PPI networks whose edges have a confidence value above a user-defined threshold *c*_min_. We denote by *E*(*v*_1_,*v*_2_) the *E*-value of proteins *v*_1_ ∈ *V*_1_ and *v*_2_ ∈ *V*_2_ obtained by an all-against-all sequence alignment. Typically, *G*_1_ is a smaller query network such as a specific pathway of interest, and *G*_2_ is a large species-specific PPI network.

A *network alignment* is a partial injective function *a*:*V*_1_ → *V*_2_ with the additional requirement that if *v*_1_ ∈ *V*_1_ is aligned then *a*(*v*_1_) ∈ {*v*_2_ ∈ *V*_2_ ∣ *E*(*v*_1_,*v*_2_) ≤ *E*_max_}. That is, every node *v*_1_ ∈ *V*_1_ is related to at most one node *v*_2_ ∈ *V*_2_ with *E*-value *E*(*v*_1_,*v*_2_) below a pre-specified cut-off *E*_max_ and vice versa. We score the topology component of an alignment *a* as follows

$$t(a)=\frac{1}{\min\{|E_{1}|,|E_{2}|\}}\underset{\text{uv}\in E_{1}}{\sum }w(u,a(u),v,a(v))$$

with

$$w\left(u,a(u),v,a(v)\right)=\left\{\begin{array}{ll} 1 & \;\text{if}\;\left(a(u),a(v)\right)\in E_{2},\\ 0 & \;\text{otherwise}. \end{array}\right)$$

This score is also known as *edge correctness* and denotes the fraction of edges from the smaller query network that have been aligned. The problem of global pairwise network alignment is to find the highest-scoring alignment. Should there be several alignments with the same maximum edge correctness, we would prefer the alignment with the highest overall bit score as obtained by an all-against-all sequence alignment. We achieve this in the following way. Let *b*(*v*_1_,*v*_2_) ∈ [0,1] be the normalized bit score of aligning protein *v*_1_ ∈ *V*_1_ with protein *v*_2_ ∈ *V*_2_. The total score of an alignment *a* is then

(1) $$\begin{array}{ll} \;s(a)=t(a) & +\frac{1}{1+\min\{|E_{1}|,|E_{2}|\}\cdot \min\{|V_{1}|,|V_{2}|\}}\\ & \cdot \underset{u\in V_{1}}{\sum }b(u,a(u)). \end{array}$$

That is, the score component is ensured to be strictly smaller than the score contribution of one conserved edge. Therefore ties among alignments with the same edge correctness are broken in favor of those with the highest overall bit score.

We use NATALIE to compute alignments with maximum total score. A specific feature of NATALIE is that any identified solution comes with an upper bound on the optimal score value. In the NATALIEQ setting with small query networks, the upper bound equals the score of the alignment found, thereby proving its optimality. The identified alignment is not necessarily optimal if there is a gap between the score and the upper bound. In that case the relative size of the gap provides a bound on the error due to suboptimality. In a recent study [4] on aligning PPI networks of six different species, NATALIE was compared to state-of-the-art network alignment methods, evaluating the number of conserved edges as well as functional coherence of the modules in terms of Gene Ontology annotation. The study established NATALIE as a top network alignment method with respect to both alignment quality and running time.

### Databases

We currently provide eight model species from STRING [1] and IntAct [8] as target databases. We added textual descriptions to the protein IDs. For the STRING networks, these descriptions are available as a separate publicly available download. We retrieved the protein descriptions for the IntAct networks by cross-referencing the IntAct UniProt identifiers with the Swiss-Prot and TrEMBL databases [9]. To allow NATALIEQ to take protein sequence information into account, we stored the amino acid sequences of the proteins in separate FASTA files per network. We retrieved these sequences from the STRING and IntAct databases. The target databases will be updated upon new releases of STRING and IntAct.

### Processing

NATALIEQ computes a network alignment in a two-step fashion implemented in a Perl wrapper script. First, the wrapper invokes BLAST [10,11] to create pairwise protein alignments between the sequences corresponding to the nodes of the query and target network. Next, the wrapper invokes NATALIE[3,4] for different *E*-value cut-offs *E*_max_ ∈ {0,10^-100^,10^-50^,10^-10^,1,10,100}. Each cut-off *E*_max_ imposes restrictions on the allowed pairings, that is, only pairs (*u*,*a*(*u*)) with *u* ∈ *V*_1_ whose *E*-value is at most *E*_max_ are allowed. During these computations, which take a few minutes for a typical network query, the user is updated about the progress and may bookmark the unique web page for this run or leave an e-mail address to be notified upon completion.

## Results and discussion

### Web server

The input of NATALIEQ consists of a query network that can be in several formats: a simple edge list format, Cytoscape’s SIF format, IntAct’s MITAB format or STRING’s text-based format. The input file format is automatically detected. Optionally, the user can provide a FASTA file containing the protein sequences corresponding to the network nodes. In case no FASTA file is supplied and the node labels correspond to UniProt, RefSeq or GI identifiers, the corresponding sequences are retrieved automatically from the NCBI Protein database [12]. The user can select one of two well-known protein interaction databases (IntAct or STRING) and one of currently eight model species as target network. Options are the score function and the confidence threshold *c*_min_. We support two score functions: *topology*, which is the scoring function as defined previously, as the default option, and *sequence only*, which results in the best network alignment in terms of sequence similarity, disregarding topological information.

**Figure 1:**
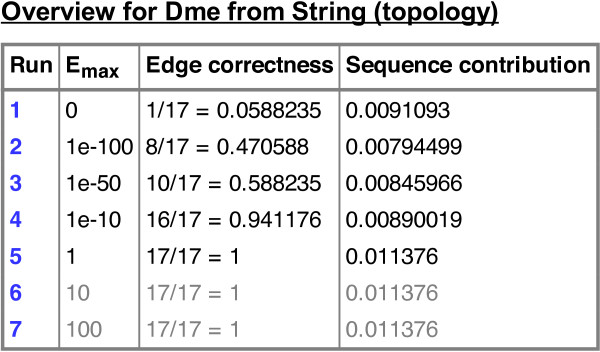
**NATALIEQcomputation overview of the alignments of the Wnt query network against the target PPI network (STRING) of** ***D. melanogaster* using the** ***topology* score function.**

**Figure 2:**
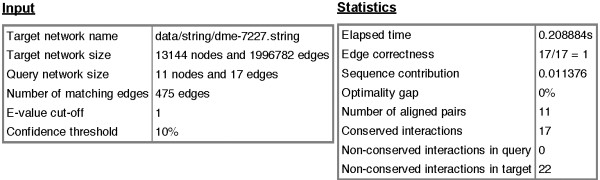
**NATALIEQsummary statistics for run number 5 (*E***_**max**_** = 1).** Alignment of the Wnt query network against the target PPI network (STRING) of *D. melanogaster* using the *topology* score function.

**Figure 3:**
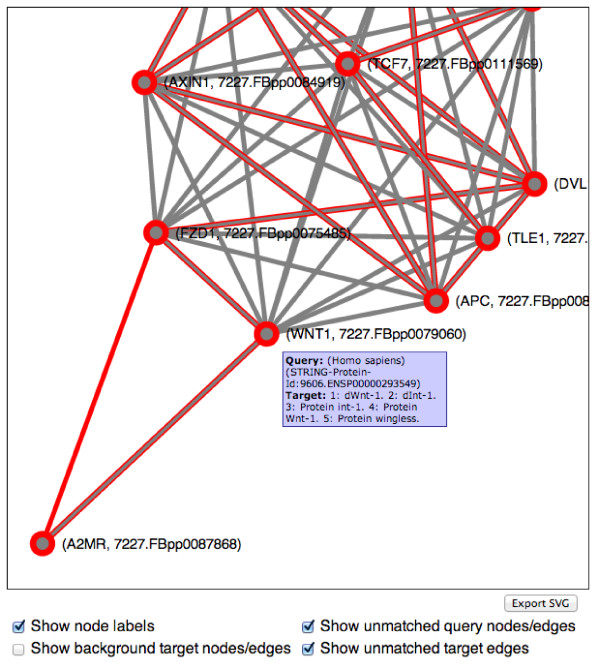
**NATALIEQinteractive visualization component showing the alignment of the Wnt query network (red) with the target PPI network (STRING, grey, matched part shown) of** ***D. melanogaster* using the** ***sequence only* score function at** ***E*-value cut-off 1.** The purely red edges, for example, (FZD1, A2MR), hint at interactions that have been missed by the alignment. See also Figure 4, bottom table. The tool-tip appears when hovering over the nodes.

The output page first gives an overview of the results for the different *E*-value cut-offs (Figure 1). The user can select a result for detailed inspection. Interesting results to inspect are, for example, the one with best sequence similarity among the top-scoring topological similarities or the one with best topological score at lowest *E*-value cut-off. The detailed view starts with summary statistics about the input networks and the computational process (Figure 2). It then displays an interactive network alignment visualization using the Javascript D3 library ( http://mbostock.github.com/d3/), which is a data-driven framework for information visualization. The visualization (Figure 3) shows the aligned part of the two networks, overlaying nodes and links using red color for the query and grey for the target network. Thus, a matched query-target node or link pair will be colored in both red and grey. This interactive network visualization shows the user which parts of the query and target networks are matched. Hovering over nodes and links displays tool-tips with protein names and descriptions and link confidence, respectively, and allows for a quick overview of the alignment. If the user clicks on a node, information about that node is shown in a separate table, which in addition to the protein names and descriptions includes the bit score and *E*-value of the BLAST pairwise alignment and a hyperlink to the original database for more information about the target protein. The interface allows for a more detailed analysis by toggling the visibility of node labels, background target nodes and edges, unmatched query nodes and edges, and unmatched target edges.

**Figure 4:**
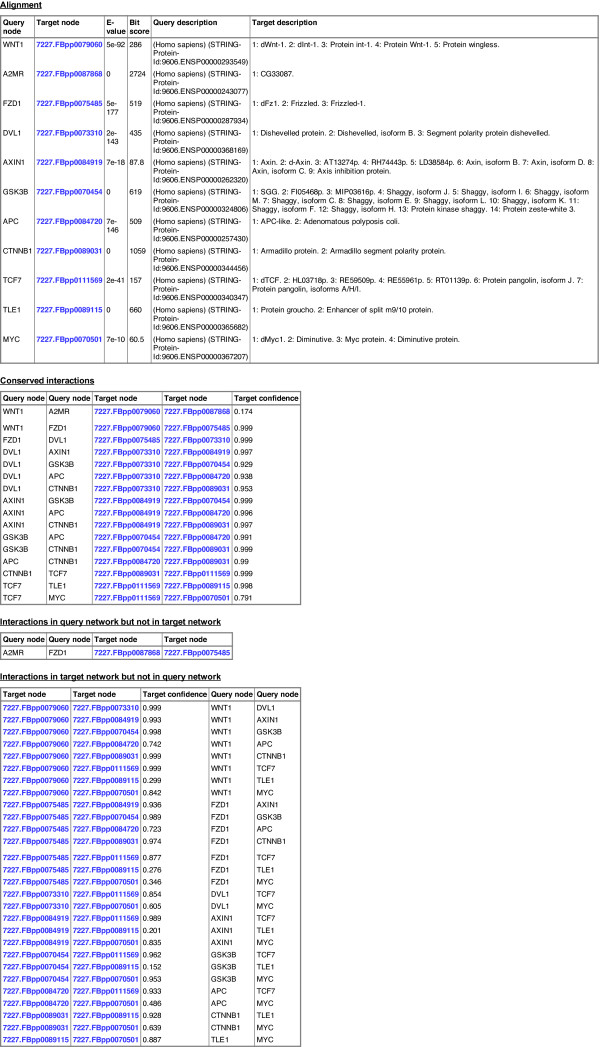
**NATALIEQalignment tables for the alignment of the Wnt query network against the target PPI network (STRING) of** ***D. melanogaster* using the** ***sequence only* score function at** ***E*-value cut-off 1.** Blue entries are links to the STRING database.

In addition, the detailed view shows tables containing aligned query-target nodes, edges conserved in both query and target network, edges in the query network that remain unaligned, and unaligned edges in the target network whose incident nodes are aligned (Figure 4). The interactive visualization can be exported to a static SVG file and the user can download the alignment and the interaction tables for further off-line analysis. We support Cytoscape [13] by providing Cytoscape-compatible files containing the entire alignment and query network as well as matched parts of the target network.

### Case study: Wnt signaling pathway

To illustrate the capabilities of NATALIEQ, we consider a biological case study involving the Wnt signaling pathway whose abnormal signaling has been associated with cancer. This pathway is initiated by binding of secreted Wnt signaling proteins to the cell surface receptors Frizzled and LRP. This causes the activation of the signaling protein Dishevelled, which in turn inhibits the assembly of the degradation complex GSK-3 *β*/axin/APC/ *β*-catenin. As a result, the degradation of *β*-catenin is prevented causing it to accumulate in the nucleus. There, *β*-catenin forms a complex with LEF-1/TCF thereby displacing Groucho. The newly formed complex induces the transcription of various Wnt target genes, including c-myc which is a proto-oncogene encoding for a protein involved in cell growth and proliferation [14].

We manually constructed a PPI network of the pathway by using a subset of the proteins involved, namely WNT1, A2MR (LRP1), FZD1 (Frizzled-1), DVL1 (Dishevelled), AXIN1, GSK3B, CTNNB1 (*β*-catenin), APC, TCF7, TLE1 (Groucho), and MYC. For each of these proteins, we obtained their respective sequences from the STRING database. The edges we used correspond to the interactions described above. The query network consists of 11 nodes and 17 edges and is available as the example network file on the main page of NATALIEQ.

As a first sanity check, we queried against the human PPI network from STRING with link confidence threshold *c*_min_ = 0.1. For all *E*-value cut-offs, NATALIEQ found the optimal alignment where indeed all interactions are present and all query proteins are aligned with their identical counterparts in the human network as we could verify from the descriptions and interaction tables in the output.

For our next experiment, we used the PPI network of *D. melanogaster* as target. See also Figures 1– 4 for an illustration. To study whether topological information improves comparative analysis, we compare the results of NATALIEQ using both the *topology* and *sequence only* score functions. We see that in the resulting *sequence only* alignments for *E*-value cut-offs larger than 10^-10^ one interaction of the query network is not mapped. This is the interaction between A2MR and FZD1. The counterpart of FZD1 in the sequence only alignment is FBpp0075485 with a bit score of 519 (*E*-value: 5 · 10^-177^). The web server also provides the BLAST output, which shows that FZD1 is indeed sequence-wise most similar to FBpp0075485. NATALIEQ with the *topology* score function at *E*-value cut-offs larger than 10^-10^ is able to match all (17) query interactions and pairs FZD1 and FBpp0077788 with a bit score of only 150 (*E*-value: 6 · 10^-38^). Although the bit score is less than the one obtained in the sequence-only alignment, the interaction A2MR–FZD1 is now present in the target network and has a normalized confidence of 0.172. So using NATALIEQ, we find that FZD1 may functionally be more related to FBpp0077788 than its sequence-wise most similar counterpart FBpp0075485. This hypothesis is corroborated by UniProtKB/SwissProt annotation indicating that the protein FBpp0077788 contains a Frizzled domain. Running the same example using the NetAligner web server [7] results in only 5 conserved interactions using default settings.

This example illustrates how NATALIEQ can facilitate the transfer of functional annotation across species. For instance, we could transfer functional annotation concerning the Wnt pathway between the human and fly networks by using the alignments we obtained.

## Conclusions

We developed NATALIEQ, a web server for global pairwise network alignment of a pre-specified query PPI network to a selected target network. The underlying alignment method computes alignments with a worst-case bound on their quality. For the biological query networks we considered, the optimality gap was closed and provably optimal alignments with respect to the used score function were thus found. The user can quickly get an overview of the alignment through the interactive visualization, where conserved and non-conserved interactions are easily visible.

Currently, we support eight different target species from both STRING and IntAct. NATALIEQ is extendible, and we will add more target networks in the future. In addition, we plan to exploit the general applicability of the underlying NATALIE method by facilitating the identification of network motifs through more sophisticated query networks where nodes are labeled by GO terms and edges are labeled by different interaction types, such as inhibition and activation.

## Availability and requirements

• **Project name:** NatalieQ

• **Project home page:**http://www.ibi.vu.nl/programs/natalieq/

• **Operating system(s):** Platform independent

• **Programming language:** PHP and Perl

• **Other requirements:** modern web browser (Internet Explorer 9 or higher, Firefox, Chrome or Safari)

• **Any restrictions to use by non-academics:** no license required

## Competing interests

The authors declare they have no competing interests.

## Authors’ contributions

NatalieQ was conceived by all authors. MEK, BWB and GWK designed and implemented the web interface and processed the data. All authors contributed to the writing of the manuscript and approved the final manuscript.

## Authors’ information

Mohammed El-Kebir and Bernd W Brandt are joint first authors and both authors contributed equally.
